# Laboratory Diagnosis of Lassa Fever, Liberia

**DOI:** 10.3201/eid1606.100040

**Published:** 2010-06

**Authors:** Marcus Panning, Petra Emmerich, Stephan Ölschläger, Sergiusz Bojenko, Lamine Koivogui, Arthur Marx, Peter Clement Lugala, Stephan Günther, Daniel G. Bausch, Christian Drosten

**Affiliations:** Bernhard Nocht Institute for Tropical Medicine, Hamburg, Germany (M. Panning, P. Emmerich, S. Ölschläger, S. Günther, C. Drosten); United Nations Mission in Sierra Leone, Freetown, Sierra Leone (S. Bojenko); Program on Hemorrhagic Fevers, Conakry, Guinea (L. Koivogui); World Health Organization, Geneva, Switzerland (A. Marx); World Health Organization, Monrovia, Liberia (P.C. Lugala); Tulane University Health Sciences Center, New Orleans, Louisiana, USA (D.G. Bausch)

**Keywords:** Lassa fever, Liberia, laboratory diagnosis, reverse transcription–PCR, viruses, sensitivity, letter

**To the Editor:** Lassa fever is endemic in West Africa, with <300,000 Lassa virus (LASV) infections occurring annually (*1*). Persons on humanitarian missions and peacekeeping forces in regions comprising Sierra Leone and Liberia are at risk for Lassa fever (*2*–*4*). Reliable laboratory diagnosis, particularly in acute cases, is crucial for triage, implementation of barrier nursing, and contact tracing, as well as for initiation of treatment with ribavirin. Reverse transcription–PCR (RT-PCR) is routinely used for confirmation of cases, but few proven assay formulations are available, and these have not been evaluated on larger cohorts of patients (*5*).

We summarize our experiences from testing 184 patients from Liberia with suspected cases of Lassa fever with the most widely used LASV-specific RT-PCR assay (*6*). Patients were suspected of having Lassa fever on clinical grounds by physicians of the United Nations peacekeeping troops and other international relief organizations. Patients included local citizens as well as members of the mentioned organizations. EDTA-plasma samples or serum specimens packed on ice were sent to our laboratory in Hamburg, Germany, by international airfreight, taking 4–7 days for shipment. Information on clinical signs and symptoms or outcome was generally not available.

Conventional RT-PCR specific for the glycoprotein precursor gene was conducted as described (*7*). RT-PCR results positive for LASV was seen in 35 (19%) of 184 patients. Median time between onset of symptoms and sampling was 7 days. Median time from reception of samples to final RT-PCR or culture results was 1 day and 4 days, respectively.

Although samples were usually thawed upon reception, all samples positive by RT-PCR were also positive by cell culture. Three additional samples were positive by culture but repeatedly negative by RT-PCR in serum. PCR inhibition had been excluded in these samples by testing duplicates of the same samples spiked with LASV RNA. The associated culture supernatants tested positive by the same RT-PCR, as well as immunofluorescent antibody microscopy. A 522-bp fragment of the glycoprotein precursor gene spanning the entire RT-PCR fragment (334 nt) was amplified for sequence analysis with primers S36 (*6*) and LVS526 (5′-aaaatcgcagctcattgcctcata-3′).

In each of the 3 individual isolates, several mismatched nucleotides at the binding site of the antisense primer S80 (*6*) were observed. To obtain a clearer picture of the relevance of sequence variability, we randomly selected and sequenced 9 additional samples positive by RT-PCR samples described in this study (Table).

**Table:** Laboratory results and primer sequences on 12 patients with Lassa fever, Liberia*†

Strain designation‡	Deviations from sequence (5′ → 3′)‡	Original primers (*6*)	Modified primers	Cell culture	Log_10_ RNA copies/mL	Anti-LASV IgM titer‡	Anti-LASV IgG titer‡
	TGCACAAAGAACAACAGTCATCATTATAT						
129/05	---T----A-----T-----C--------	–	+	+	3.20	Neg	<10
UN133	---T----A-----T-----C--------	–	+	+	3.99	Neg	<10
121/1580	--T-----A-----------C--C-----	–	+	+	4.14	Neg	<10
127/05	--------A--------------------	+	+	+	6.22	Neg	<10
4094/05	--------A-----------------C--	+	+	+	4.53	Neg	<10
Lib3800	--T-----------------C--------	+	+	+	4.90	160	5,120
Lib88	--T-----------------C—-C-----	+	+	+	7.49	40	20
Lib90	--T-----------------C—-C-----	+	+	+	5.17	Neg	<10
295/06	--T-----------------C—-C-----	+	+	+	5.41	80	640
383/06	--------------------C--C--C--	+	+	+	4.38	40	<10
120/06	--------A--------C--C-----C--	+	+	+	4.50	Neg	<10
174/06	-----------TG-T--C--C--C--C--	+	+	+	5.02	320	640

*LASV, Lassa fever virus; Ig, immunoglobulin. †Primers sequences are compared to that published by Demby et al. (*6*) (reverse primer binding sites). Virus isolation was done on Vero cells in a BioSafety level 4 laboratory. Serologic testing for anti-LASV IgM and IgG was performed by using the indirect immunofluorescent assay. ‡From reference strain AY628203, position 3092–3064 = reverse primer binding site in (*6*); note that the forward primer is not analyzed because it is located in the conserved stem-loop structure of LASV small segment RNA.

Mismatched nucleotides were observed with all 12 strains. Up to 3 nucleotide mismatches apparently did not prevent amplification, whereas 4 positions appeared to be a critical threshold for PCR failure (nevertheless, 2 samples with 4 and 7 mismatches did amplify). Primer S80 (*7*) was modified with respect to mismatches, and RT-PCR was repeated on the 3 plasma samples that initially were negative by RT-PCR. As expected, they now were positive.

For an exact determination of virus RNA concentrations in the 12 samples, 12 individual probe-based real-time RT-PCRs were designed upon determined virus sequences (Table). Mean RNA concentration in all samples was 8.13 × 10^4^ copies/mL. Notably, samples that had initially tested false negative showed significantly lower mean virus RNA concentrations than the overall mean (5.8 × 10^3^ cop/mL; p<0.05 by *t* test). The limit of detection of the screening RT-PCR was 2,500 copies/mL for a perfectly matched template (*7*), making it clear that there was not a huge buffer (reserve) in sensitivity for the screening assay.

A total of 17 (9.2%) of 184 samples displayed immunoglobulin (Ig) M or IgG antibodies to LASV, or both types of antibodies. LASV-specific antibodies were detectable in only 11 (29%) of 38 cell culture–positive samples, and 6 samples yielded LASV-specific antibodies with no concomitant positive result by cell culture (Table). Four of these displayed IgG only, indicating previous rather than acute infection. The 3 false-negative RT-PCR samples were negative for IgM and IgG.

Our study underscores the utility and shortcomings of RT-PCR diagnostics for Lassa fever. Although RT-PCR is an appropriate diagnostic tool, it may fail to amplify strains even with limited sequence deviations, as already cautioned in early presentations of methods (*6*). Failure to amplify divergent LASV strains has been observed (*2*,*8*), and considerable sequence diversity in LASVs has been noted even within relatively constricted geographic areas (*9*). Current oligonucleotide binding sites are not conserved enough for diagnostic application without continuous assessment and revision of primer sequences. Cell culture remains the diagnostic standard for LASV (*10*), but this technique remains unavailable in many Lassa fever–endemic areas of West Africa. If virus isolation is not feasible, diagnostics should include RT-PCR, combined with enzyme immunoassay antigen detection (which may be less susceptible to false negatives due to strain variation [*10*]), or at least 2 complementary LASV RT-PCR assays. Serologic testing for IgM and IgG antibodies by enzyme immunoassay or immunofluorescent antibody assay should also be performed. Although not currently feasible in West Africa, external quality control should include virus culture. Physicians should be aware of the limitations of laboratory diagnostic assays for LASV.
